# Development and Application of a Program for Reinforcing Global Health Competencies in University Nursing Students

**DOI:** 10.3389/fpubh.2020.00263

**Published:** 2020-06-30

**Authors:** Won Ju Hwang, Hyun Hee Jo

**Affiliations:** College of Nursing Science, Kyung Hee University, Seoul, South Korea

**Keywords:** academic global health programs, global health capability, global leadership, global citizenship, critical thinking

## Abstract

**Purpose:** With globalization, the importance of global health is being stressed. Although nurses are indispensable healthcare professionals, programs to develop nurses with global health competencies and relevant studies are rare. Accordingly, this study was designed to develop a program to increase global health competencies and global leadership in nursing students and test its effect.

**Methods:** A single group pre- and post-test study design was used. A 13-weeks program was developed and implemented with 204 students. Data were analyzed using paired *t*-test. The program to promote global health competencies was designed to improve nursing students' knowledge of global health, global leadership, global health competencies, critical thinking ability, and student-student partnership.

**Results:** Global health competencies (*t* = −19.96, *p* < 0.001), self-assessed global leadership (*t* = −7.67, *p* < 0.001), and critical thinking ability (*t* = −7.67, *p* < 0.001) all significantly increased.

**Discussion:** The study findings of increased global health competencies and global leadership in nursing students after participation in the program indicated ways in which global health competencies of nurses need to be developed. Therefore, the following aspects should be considered. First, nursing educators should understand the need and importance of global health education and accordingly, current nursing curriculums should be revised to include courses about global health. A systematic course of studies about global health should be developed and implemented based on discussions among experts and researchers. Second, as future health care professionals, nursing students should take an interest in global health problems and recognize various issues that need to be solved beyond borders between countries. To develop global health competencies, various efforts and systematic curriculum revisions are necessary.

## Introduction

As international interdependency is increasing due to advancements in globalization, information, and air traffic, the world is experiencing an increase in human mobility; hence, it is important to make efforts to address infectious and non-infectious diseases and health inequalities (1). Examples that clearly represent such problems include the recent outbreak of COVID-19 that started in Wuhan, China, in 2019, Middle East Respiratory Syndrome (MERS), Ebola virus, new types of flus such as bird flu, and Severe Acute Respiratory Syndrome (SARS). The current rapid spread of COVID-19 not only causes economic and societal problems but also prompts a variety of measures to be taken such as travel restrictions for the citizens of the countries with an outbreak, inducing the phenomenon of a problem within a country becoming a global health issue, and a global health problem becoming a national issue, consequently resulting in international disputes and conflicts globally.

The awareness of the importance of global health is spreading among public health experts, policymakers, and practitioners in many aspects (2). Of them, nurses are a primary group contributing to global health management and their role should be expanded further. It has been pointed out that to continue improving global health, the quality of health workers, particularly professionalism, and leadership skills, should be enhanced (3). The size of investment in public health projects has steadily increased, and the Global Advisory Panel on the Future of Nursing stresses nursing education, practice, research, and leadership to develop and sustain actions and solutions to achieve the goal (4). The International Council of Nurses (ICN) expounds that nurses are professionals who promote human health thus should have an understanding of social determinants of health like poverty, education, stress, employment and occupation, and food safety, which are areas where nurses can apply their knowledge and experiences, and that quality of life of individuals, families, communities, and citizens of a country will improve through such nursing activities (4, 5).

In university and public health science in particular, students' interest in global health is increasing. With increasing global interconnectedness, the worldwide need to address difficulties with respect to health equalities and health service distribution is also increasing (6). Regarding this need, some nursing schools such as the University of California, John Hopkins University (JHU), and the University of Pennsylvania in the United States expanded opportunities for global health research and education (6, 7). For advancement in global health, it is essential for health professionals including nurses to have global health competencies (8, 9), and research and education in the field are in dire need. Gimbel et al. (6) reviewed global health programs offered in schools of nursing in the US and based on the review, established a foundation for the Research, Education, Policy, and Partnership (REPP) framework. Using the framework, academic entities can ensure that core areas are addressed when developing and implementing strategies to promote effective nurse participation in global health research, education, and policy development through effective partnership. The researchers mentioned that through the REPP framework, it is possible to form an overarching consortium between nursing and non-nursing organizations and reinforce interdisciplinary coordination. Gimbel et al. (6) summarized the programs in the top 10 nursing schools in the US in 2017. To examine the current status of global health education provided in the US nursing colleges, JHU offers “International placements (service-learning, research training programs, fellowships, and practicums abroad), International Visiting Scholar Program, consultation services for foreign schools of nursing, Coverdell Fellows program (a graduate school program for returned Peace Corps Volunteers), and is a key partner in the JHU Center for Global Health (collaboration between all of the JHU schools that harnesses the expertise of its dedicated health and medical professionals to address a myriad of global health challenges)” (p. 120). The University of Pennsylvania offers “Global Women's Health Research Center, a global health nursing (GHN) minor, Coverdell fellows, study abroad, research partnerships, and international visitors” (p. 120), while the University of California system and Duke University offer “GHN fellowship, international collaborations/visitors, GHN minor, GHN Bootcamp, GHN clinical scholars and so on” (p. 120).

Global health is an essential competency for nurses and its importance has continuously increased. In Korea, though nurses and nursing students are required to have global health competencies, the curriculum for global health competency is not standardized. Particularly, relative to the level of nursing in Korea, nurses' understanding and use of the official development assistance (ODA) and global health is very low. Education provided by nursing colleges to improve global health capabilities is offered in academic seminars and special lectures therefore, there are few opportunities to get systematic education. Few educational institutions in Korea offer global health education program because of the cost burden for the training and education (international dispatch, manpower, goods support, etc.) (10). To develop nurses who can help solve global health problems in the future, a revolutionary change should occur in education and educational institutions. One goal of education should be to improve integrative ability so as to expand the scope of practice to a setting in which resources are lacking but the demand is high (11). In developing nursing students into future global health workers, it is necessary to train them to be capable of understanding the distinctive features of global health programs, global health standards, and in directing and evaluating them. Accordingly, it is necessary to have a global health education program if such capabilities are to be developed (10). Nursing educators should actively research and develop methods to enhance global health competencies in nursing students and nurses, and these students and nurses should be able to evaluate and apply the competencies (12). Furthermore, nurse educators should consider critical thinking—the process of thinking and reflection—that pervades all human activities and is crucial to make independent judgement, decisions, and exclusion in practice (13). The goals of a systematic curriculum for global health should aim for nursing students to play the role of global health practitioners and develop global leadership (10). Additional goals are to reinforce the new area of global health and nursing science, support global health education for future nursing scientists, and promote nurses' participation in the formation of global health policies (6).

Accordingly, for this study we wanted to develop and implement a systematic and integrated program for reinforcing global health competencies in nursing students and test the program's effect. We hypothesized the study findings would provide a foundation based on which global health programs could continue to improve and serve as basic data in creating nursing college courses on global health to develop nursing students' global leadership and global health competencies.

The purposes of this study were to develop a program for reinforcing global health competencies in nursing students and identify the changes in global leadership, essential global health competencies, and critical thinking in the students by comparing these factors before and after participation in the program.

## Materials and Methods

### Study Design and Subjects

The study was conducted using single group pre- and post-test design to assess the effects of the education program developed to reinforce global health competencies in nursing students and identify the changes in essential global health competencies, global leadership, and critical thinking.

To implement the global health education program, a participant recruitment flyer targeting 2nd year nursing students was posted at K University located in Seoul. The flyer contained an explanation of the study purposes and duration and informed students who wanted to participate in the study to contact the researchers. A total of 221 students were recruited for the study, and the final number of study participants was 204 after 16 were excluded (13 who did not complete the survey and three who missed six or more sessions). The sample size satisfied the minimum requirement estimated using G^*^Power 3.1, *n* = 63, under the assumptions of effect size for paired *t*-test of 0.42 (10), a significance level of 0.05, and a power of 95%.

### Development and Application of a Program for Reinforcing Global Health Competencies in Nursing Students

#### Educational Program Development

**Figure 1:**
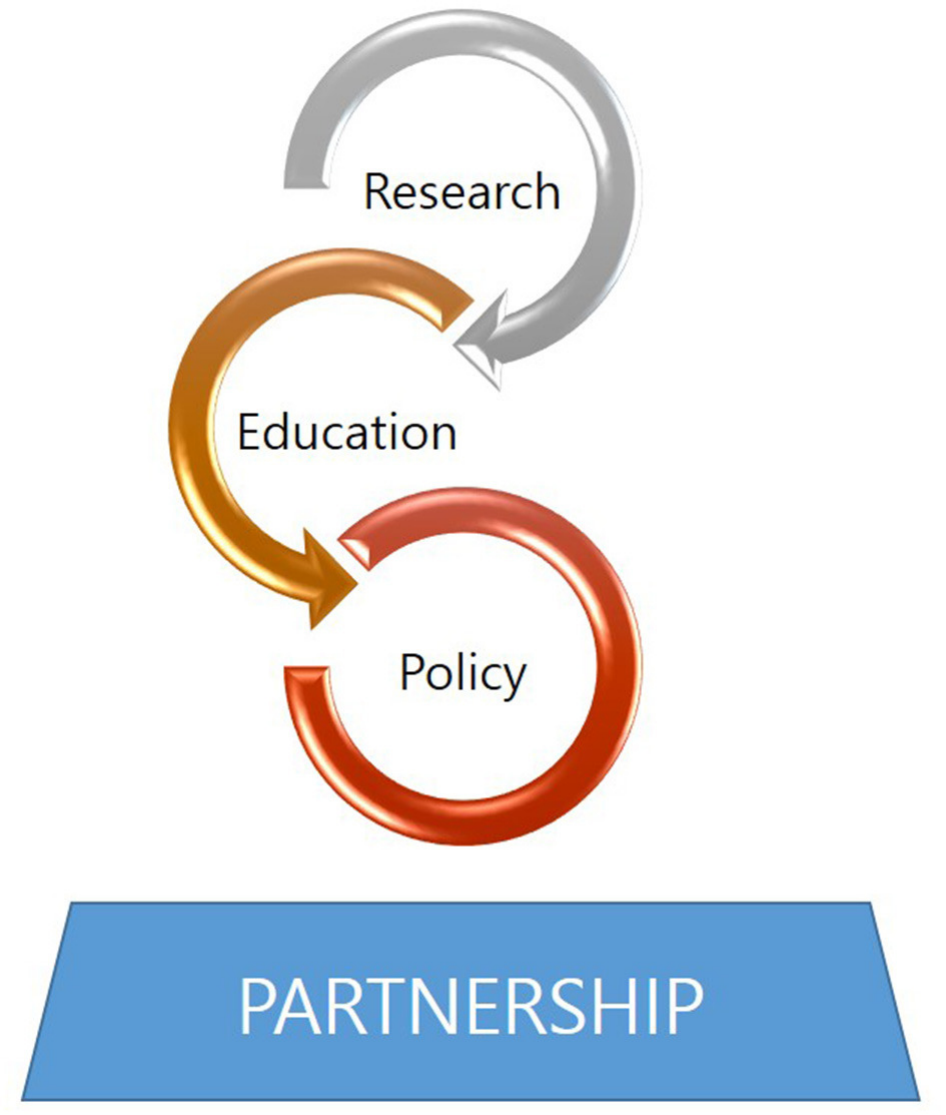
REPP framework [as cited in (6)].

The program was designed based on existing programs and essential competencies required for nursing students, which were discussed in published literature related to global health competencies. Of specific areas receiving support, public health and education constituted a large proportion, did the expansion of national support policy in ODA; hence, contents related to those areas were reflected in the program. Revisions and improvements were made to the program through numerous meetings with a nursing professor with expertise in global health and ODA. The program was created with the aim of enhancing nursing students' knowledge of global health, global leadership, global health competencies, critical thinking, and partnerships between students. The program was based on the strategy to promote successful nurse participation in global health research, education, and policy development through effective partnerships using the REPP framework (6) (Figure 1).

#### Program Application

**Figure 2:**
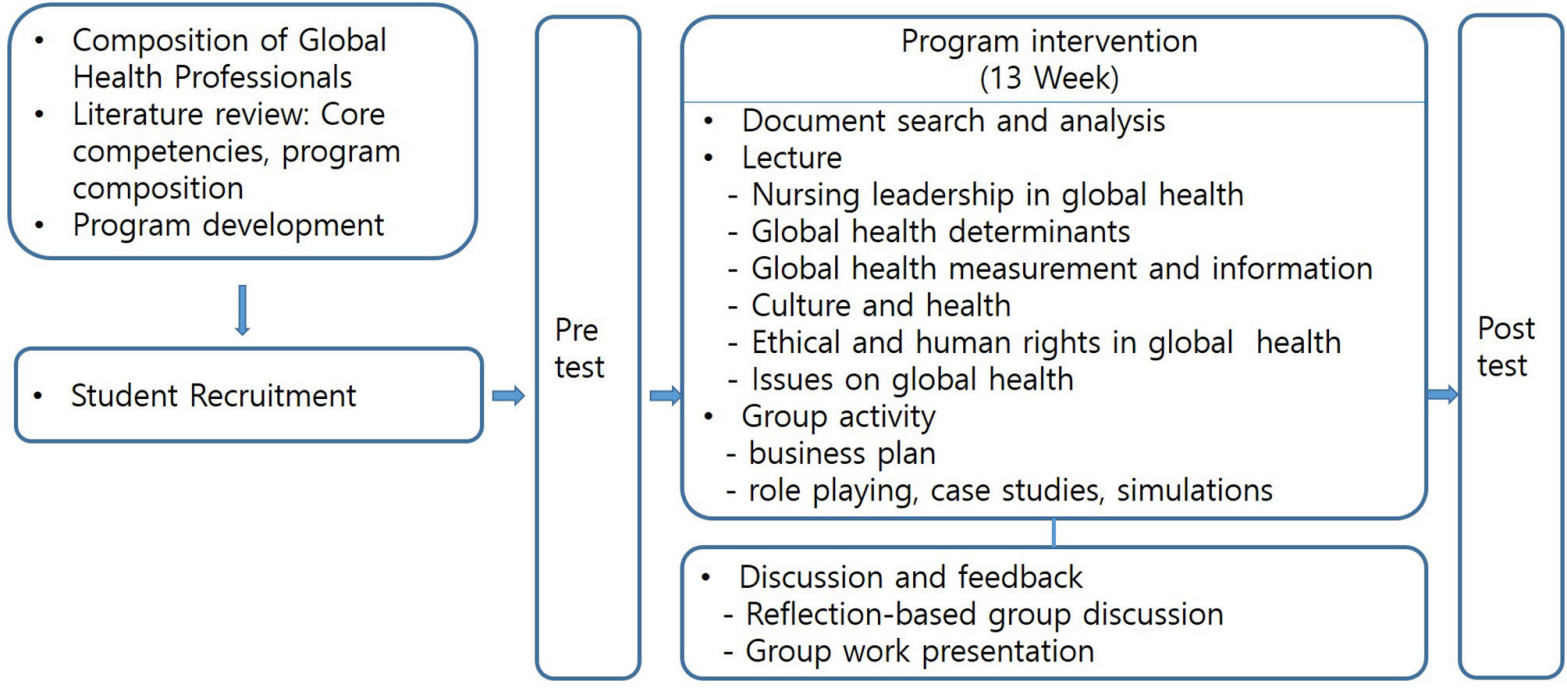
The procedure of the global health capacity building program.

The global health competency program spanned 13 weeks and was held once a week for 100 min each meeting. A total of 204 students participated in the research (Figure 2). First, we divided the group into three teams of 70 students per team in order of application. Students were then randomly assigned and divided into small groups of 5–6 students and instructed to discuss studies relevant to the topic selected for each session. Relevant specialized knowledge was provided in lectures and problem-based learning focused on the improvement of global leadership and global mindset by teaching students to have accurate knowledge and develop wide perspectives about global health. Students were also taught to improve their critical thinking by encouraging them to uncover global health problems in developing countries and identify ways to solve the problems. Autonomous team activities were conducted to encourage students to develop their leadership and teamwork abilities. Each team presented a global health project, and teachers and other teams provided feedback concerning the project's pertinence, topic, components, budget, and effectiveness. Additionally, students were instructed to have discussions to compare and evaluate the planned ODA projects against global health theories.

### Ethical Considerations

The study was approved by the Institutional Review Board (IRB) of K University, where the principal investigator was working [KHSIRB-17-074 (NA)]. Nursing students who consented to participate in the study were informed of the study purposes and the study survey was administered to only those who voluntarily consented to participate. Potential participants were also informed that they could withdraw from the study any time without penalty or loss of any benefits to which they would be normally entitled.

### Research Instruments and Data Collection

#### Essential Global Health Competencies

Essential global health competencies were assessed using the instrument developed by Lee et al. (1). This instrument can be used to assess nursing students' knowledge and understanding of essential global health competencies. The instrument consisted of a total of 24 items across the following six subdomains: global burden of disease (3); health implications of migration, travel, and displacement (6); social and environmental determinants of health (5); globalization of health and healthcare(3); healthcare in low-resource settings (5); health as a human right and development resource (2). The items were rated on a 5-point Likert scale, and the higher the score, the higher the knowledge, and understanding of essential global health competencies. Cronbach's α-values of the subdomains ranged from 0.73 to 0.90 in Lee et al. (1), and Cronbach's α in this study was 0.94.

#### Global Leadership Competency

Changes in global leadership were measured using the instrument Nam (14) revised and validated in a study conducted with college students. The instrument contained a total of 18 items across five subdomains: global mindset, open-mindedness in the attitude toward diversity, global network, outcome improvement skills, and basic attitudes, and measured perceived importance, and self-assessed level of global leadership. The items were rated on a 5-point Likert scale, and the higher the score, the higher the level of the global leadership competency. Cronbach's α was 0.83 in the study conducted by Nam (14) and 0.92 in this study.

#### Critical Thinking

Critical thinking was measured with the instrument developed by Yoon (15). It is a personal tendency to judge oneself in terms of problem-solving and decision-making in personal or professional work (16). The instrument consisted of 27 items rated on a 5-point Likert scale across the following seven subdomains: sound skepticism, intellectual fairness, objectivity, systematicity, watchfulness, intellectual passion/curiosity, and confidence. The higher the score, the stronger the critical thinking ability. Cronbach's α was 0.84 in the study by Yoon (15) and 0.81 in the present study.

#### Perception of ODA

To assess awareness of ODA, questions, and categories from the 2014 and 2016 surveys of citizens' perception of ODA were reviewed. Eight items were constructed from items common to the two surveys and iterms deemed necessary for the present research. The example of the item is “assessment on the contribution of Korea's ODA” (17, 18). Cronbach's α for this set of items was 0.74 in this study.

#### Data Analysis

Data were analyzed using SPSS/WIN21.0. Participants' general characteristics and perception of ODA were examined by computing frequencies and percentages, and pre- and post-test changes in global health competencies, global leadership, and critical thinking ability (disposition) were determined using paired *t*-tests.

## Results

The mean age of the participants was 21.4 ± 1.18 years, and 85.8% were female. The majority (61.3%) of the participants did not have experience in participating in an international cooperation activity, but 26.5% had visited a developing country. Only 13.2% indicated they had received education about international cooperation and 21.6% reported being aware of ODA in Korea. Thus, more than a half the participants had neither knowledge of international cooperation nor experience participating in international cooperation activities. However, 78.5% of the participants thought that Korea contributed to international assistance and 91.5% agreed with providing international assistance. The top reasons for the agreeing Korea should participate in international assistance were to help eradicate poverty and disease, and contribute to peaceful coexistence of humankind (58.3%) and Korea's past experience of receiving international assistance (24.5%) (Table 1).

**Table 1:** Characteristics of participants and perception of ODA (*N*= 204).

**Characteristics**	**Categories**	***n*(%)**	**Mean ±*SD* (Min-Max)**
Sex	Male	29 (14.2)	
	Female	175 (85.8)	
Age (years)	20 and younger	15 (7.4)	21.4 ± 1.18 (19–28)
	21–25	185 (90.6)	
	26 and older	4 (2.0)	
Satisfaction with Major	Dissatisfied	11 (5.54)	
	Neutral	76 (37.3)	
	Satisfied	117 (57.4)	
Religion	Yes	83(40.7)	
	No	121 (59.3)	
Having experience of participating in an international cooperation activity	Yes	79 (38.7)	
	No	125 (61.3)	
Having visited a developing country	Yes	54 (26.5)	
	No	150 (73.5)	
Having received education about international cooperation	Yes	27 (13.2)	
	No	177 (86.8)	
Awareness of Korea's international assistance	Aware	44 (21.6)	
	Not aware	160 (88.2)	
Level of contribution by Korea's ODA	High	13 (6.4)	
	Somewhat high	147 (72.1)	
	Low	38 (18.6)	
	None	4 (2.0)	
Opinion on Korea's official development assistance	Agree	188 (91.5)	
	Disagree	17 (8.5)	
Reason for agreeing with Korea's ODA	Contribution to world peace	119 (58.3)	
	Experience of receiving assistance	50 (24.5)	
	International status	11 (5.4)	
	Opportunity for Korean businesses to enter other countries	2 (1.0)	
Reasons for disagreeing with Korea's	Korea is not rich enough	8 (47.0)	
official development assistance (in	It does not benefit Korea	4 (23.5)	
case of disagree of ODA)	It does not benefit the developing countries	2 (11.7)	
	It is another country's problem	3 (17.6)	

*ODA, official development assistance*.

Changes between before and after participation in the program for reinforcing nursing students' global health competencies are presented in Table 2. The post-test score for global health competencies was 3.67 ± 0.63 points, which was significantly higher than the pre-test score, 2.57 ± 0.57 (*t* = −19.96, *p* < 0.001). Among the subdomains, global burden of disease increased the most (1.54 ± 0.88), while social and environmental determinants of health increased the least (0.84 ± 0.92).

**Table 2:** Comparison of Pre- and Post-test (*N*= 204).

**Variables**	**Pretest (M ±*SD*)**	**Posttest (M ±*SD*)**	***t***	***p***
Global health competencies for nursing students	2.57 ± 0.57	3.67 ± 0.63	−19.96	<0.001
Global burden of disease	2.08 ± 0.61	3.62 ± 0.74	−25.06	<0.001
Health implications of migration, travel and displacement	2.75 ± 0.72	3.66 ± 0.67	−14.51	<0.001
Social and environmental determinants of health	3.05 ± 0.66	3.89 ± 0.69	−13.07	<0.001
Globalization of health and healthcare	2.49 ± 0.68	3.56 ± 0.70	−15.68	<0.001
Healthcare in low-resource settings	2.27 ± 0.66	3.58 ± 0.73	−19.77	<0.001
Health as a human right and development resource	2.42 ± 0.73	3.61 ± 0.80	−17.85	<0.001
Global leadership ability (importance)	4.10 ± 0.55	4.29 ± 0.59	−4.40	<0.001
Global mindset	4.11 ± 0.62	4.29 ± 0.68	−3.43	0.001
Open-mindedness in the attitude toward diversity	4.01 ± 0.65	4.27 ± 0.68	−4.59	<0.001
Global network	4.11 ± 0.62	4.28 ± 0.65	−3.00	0.003
Outcome improvement skills	4.06 ± 0.69	4.29 ± 0.66	−4.03	<0.001
Basic attitudes	4.19 ± 0.60	4.33 ± 0.64	−2.75	0.006
Global leadership ability (self-assessment)	3.15 ± 0.72	3.56 ± 0.67	−7.67	<0.001
Global mindset	2.89 ± 1.00	3.36 ± 0.81	−7.13	<0.001
Open-mindedness in the attitude toward diversity	3.34 ± 0.77	3.73 ± 0.75	−5.92	<0.001
Global network	3.10 ± 0.76	3.54 ± 0.77	−6.59	<0.001
Outcome improvement skills	2.97 ± 0.94	3.40 ± 0.83	−6.13	<0.001
Basic attitude ability	3.32 ± 0.79	3.69 ± 0.70	−5.95	<0.001
Critical thinking	3.59 ± 0.38	3.70 ± 0.43	−2.87	0.005

In regard to global leadership, the perceived importance of the competency significantly increased after the program to a mean post-test score of 4.29 ± 0.59 (*t* = −4.40, *p* < 0.001). The self-assessed global leadership also significantly increased, from the pre-test score of 3.15 ± 0.72 to the post-test score of 3.56 ± 0.67 (*t* = −7.67, *p* < 0.001). Self-assessment scores for all subdomains of global leadership significantly increased. Among them, global mindset showed the greatest increase, with an increase of 0.47 ± 0.94 points. Critical thinking ability significantly increased from the pre-test score of 3.59 ± 0.38 to the post-test score of 3.70 ± 0.43 (*t* = −7.67, *p* < 0.001).

## Discussion

Nurses are key providers in primary healthcare comprising 60–80% of the total health system workforce and provide 90% of all healthcare services (19, 20). They have an important role in helping solve present and future global health problems. However, their role in practice such as policy forums and influential decision-making institutions in global health sector is limited (21). This is because they don't have opportunity to participate in the global health competencies. Accordingly, the importance of programs reinforcing global health competencies in nursing workforce is emphasized in global health (22, 23). In the present study, a program designed to increase global health competencies and global leadership skills in nursing students was developed and implemented. After participation in the program, nursing students showed significant increases in global health competencies, critical thinking, and perceived importance, and self-assessment of global leadership skills.

Regarding the survey question of whether the participants had received information about international assistance, 21.6% responded that they had. This proportion was lower than the finding (35.0%) by World Research (18) in the “2016 survey of citizens' perception of ODA” (p. 17). The proportion of participants who agreed with the concept of providing international assistance was 91.5% in this study, which was higher than the finding (80%) of the 2016 WHO survey. However, given that the rate of agreement with the government's provision of international assistance has been steadily decreasing since 2011, it has been argued that international assistance should continue to be considered and promoted (17, 18). In the present study, only 38.7% of the participants had experience in an international cooperation activity and only 13.2% had received education about international cooperation. These findings highlight that to effectively handle global health problems, the need to educate nursing students about these types of issues is rising (6); there are few opportunities in Korea for college nursing students to get systematic education to develop their global health competencies and educational institutions offering a global health education program are very scant due to various difficulties such as high cost (10). Relative to the high level of healthcare, technological advancement, and workforce in Korea, ODA, and global health are very poorly understood. Hence, to train nursing students to be a core workforce as future global health practitioners, a system for global health education should be in place to continuously offer educational programs for reinforcing global health competencies (24). To continue to improve global health, it has been pointed out that the quality of health workers, specifically professionalism and leadership skills, should be increased (3).

Global health competencies refer to developing capabilities to conduct research and enhance an individual's practical skills to achieve health equalities by promoting human health and increasing access to health services (2, 25). As future health professionals, nursing students should take an interest in world health problems, recognize a variety of problems including medical disasters (like SARS, MERS, Ebola, and COVID-19), maternal and infant health, malnutrition, and environmental health threats as problems to be handled beyond borders between countries. Health care professionals need to have relevant competencies to deal with these health issues. After participating in the program, nursing students in this study showed significant improvement in all essential global health competencies. The program was effective in increasing basic knowledge about global health such as global health overview, understanding of cultural diversity, global health issue, ethics, and human rights. Though “the recognition of changes in public health in and outside Korea” (one of learning achievements from nursing education programs) is important, the present findings showed that the program for reinforcing global health competencies in nursing students is also important since only 13.2% had been educated about international cooperation. However, since including global health is rare in Korea's nursing education program (10, 24, 26), the country is not developing health professionals well-versed in meeting the demands of global health. To improve global health competencies in nursing students, first, teachers should recognize the importance for education for global health competencies (27, 28). Nursing educators in the US currently recognize global health competencies as a core competency of nursing college students (9) and recommend that nurse leaders should have a global perspective and mindset about health (29). Therefore, it is necessary to make continuous efforts of develop, and implement effective programs for reinforcing global health competencies and operating such programs within the nursing curriculum (10, 12).

After participation in the program, participants' knowledge of global leadership principles significantly increased in both measures of perceived importance and self-assessment of their skills. Of global leadership competencies, basic attitudes were perceived by nursing students as the most important both before and after the program. In a study with non-nursing students, basic attitudes were found to be the most important global leadership competency (14). It is believed that basic attitudes involving ethics, creativity, challenge, and global citizenship are perceived as the foremost competency needed to perform tasks in practice. Nursing students' self-assessment showed they were lacking the most in a global mindset for the subdomains of global leadership, which was consistent with the findings from previous studies (10, 14, 30). Global mindset is a knowledge-based competency that begins with an understanding of globalization and the current situation (31). Hence, to cultivate global leadership, students must be intensively trained in it through the university curriculum (17, 32, 33). In the present study, overall global leadership and all its subdomains statistically significantly increased after the program, with global mindset showing the greatest increase.

In the program, lectures were delivered to help students understand the global health environment and provide them with knowledge and viewpoints needed to cope with global health changes by predicting the changes. The current findings related to fostering a global mindset were consistent with the previous findings about the various subdomains: global mindset significantly increased, as reported in Hwang et al. (10). In that study, students listened to a lecture about ODA presented by a global health expert and other lectures by various experts and visited the WHO office in the Western Pacific Region. Moreover, researchers have reported that as international exchange activities (including overseas volunteer activities) increased, individual skills in global leadership competencies increased (30, 34). Thus, international exchange programs offering opportunities for active participation in activities should additionally be established by forming a consortium with diverse institutions (34).

Critical thinking significantly increased after participating in the program compared to before participation. It is believed that critical thinking and problem-solving abilities were required in the processes of uncovering global health problems in developing countries, seeking ways to solve the problem and evaluating other teams' projects. Critical thinking appeared to increase by having students actively engaging in problem situations. This finding is in line with previous findings that critical thinking ability increased in nursing students who conceived a public health project or planned an international volunteer learning program (10, 34). Moreover, the ability also increased as the level of self-directed learning was higher in a team-based activity (35). However, research should continuously be conducted in this area.

## Conclusion

In the present study, a program for reinforcing global health competencies in nursing college students was developed and implemented to examine the effect of the program on increasing the knowledge and of skills of nursing students about essential global health competencies, global leadership, and critical-thinking. The program lasted for 13-weeks (once a week for 100 min per meeting) and consisted of lectures by Korean experts in global health and participation in team activities. A total of 204 nursing students participated in the program. After the program, knowledge and understanding of essential global health competencies, global leadership, and critical-thinking increased significantly. The findings should be considered in two aspects. First, nursing educators should understand the need for and importance of global health education. Based on this understanding, the current curriculum should be revised to include courses about global health. A systematic course of studies about global health should be developed and implemented based on discussions among experts and the findings from diverse research studies. Second, as future healthcare professionals, nurses should take an interest in global health problems, and recognize various issues such as maternal and infant health, malnutrition, and environmental health threat as problems spanning beyond international borders. This study confirmed that various efforts and systematic curriculum are required to cultivate global health competencies in nursing students. However, the ODA tool is only intended to check the degree of recognition and experience, and a tool with acceptable psychometrics need to be confirmed. Additionally, through research, programs to improve skills beyond international health knowledge and understanding should be developed and applied. To generalize the study findings, this study should be replicated, and quasi-experimental studies should be conducted. It is suggested that nursing educators and global health experts engage in integrative efforts and increase problem recognition and problem-solving through public debates.

## Data Availability Statement

The datasets generated for this study are available on request to the corresponding author.

## Ethics Statement

The studies involving human participants were reviewed and approved by Institutional Review Board (IRB) of Kyung Hee University. The patients/participants provided their written informed consent to participate in this study.

## Author Contributions

WH and HJ participated in the design of this study, analyzed the data and interpreted the results, and wrote the manuscript. WH directed this study. All authors read and approved the final manuscript.

## Conflict of Interest

The authors declare that the research was conducted in the absence of any commercial or financial relationships that could be construed as a potential conflict of interest.

## References

[1] Lee HK Kim HS Cho E Kim S Kim J Global health competencies for undergraduate nursing students in Korea. J Korean Acad Soc Nurs Educ. (2015) 21:561–73. 10.5977/jkasne.2015.21.4.561

[2] Hwang WJ Park Y. Factors influencing the accessibility of maternal health service in cambodia. Int J Environ Res Public Health. (2019) 16:2909. 10.3390/ijerph16162909 31416153 PMC6719141

[3] Bhutta ZA Chen L Cohen J Crisp N Evans T Fineberg H. Education of health professionals for the 21st century: a global independent commission. Lancet. (2010) 375:1137–8. 10.1016/S0140-6736(10)60450-3 20362799

[4] Wilson L Mendes IAC Klopper H Catrambone C Al-Maaitah R Norton ME. ‘Global health' and ‘global nursing': proposed definitions from the global advisory panel on the future of nursing. J Adv Nurs. (2016) 72:1529–40. 10.1111/jan.12973 27062286

[5] ICN Global Nursing Leadership Institute (GNLI)™ 2020. (2019) Retrieved from https://icn.eventscase.com/EN/gnli2020/Programme_Overview (accessed 10 April 2020).

[6] Gimbel S Kohler P Mitchell P Emami A. Creating academic structures to promote nursing's role in global health policy. Int Nurs Rev. (2017) 64:117–25. 10.1111/inr.12338 28052329

[7] Archambault N Incorporating Global Health into Undergraduate Nursing Education Unpublished master's thesis, University of British Columbia, Vancouver (2010).

[8] Jogerst K Callender B Adams V Evert J Fields E Hall T. Identifying interprofessional global health competencies for 21st-century health professionals. Ann Global Health. (2015) 81:239–47. 10.1016/j.aogh.2015.03.006 26088089

[9] Wilson L Harper DC Tami-Maury I Zarate R Salas S Farley J. Global health competencies for nurses in the Americas. J Prof Nurs. (2012) 28:213–22. 10.1016/j.profnurs.2011.11.021 22818191

[10] Hwang S Kim JS Ahn H Kang SJ Development and effect of a global health capacity building program for nursing students. J Korean Acad Community Health Nurs. (2015) 26:209–20. 10.12799/jkachn.2015.26.3.209

[11] Maier CB Aiken LH. Task shifting from physicians to nurses in primary care in 39 countries: a cross-country comparative study. Eur J Public Health. (2016) 26:927–34. 10.1093/eurpub/ckw098 27485719

[12] Long T. Influence of international service-learning on nursing student self-efficacy toward cultural competence. J Nurs Educ. (2014) 53:474–8. 10.3928/01484834-20140725-02 25054475

[13] Naber J Wyatt TH. The effect of reflective writing interventions on the critical thinking skills and dispositions of baccalaureate nursing students. Nurse Educ. Today. (2014) 34:67–72. 10.1016/j.nedt.2013.04.002 23623746

[14] Nam K Importance-Performance Analysis on Global Leadership Competency-focus on Employees in Global Enterprises and College Students. Unpublished master's thesis, Hanyang University, Seoul (2011).

[15] Yoon J A study on the critical thinking disposition of nursing students-Focusing on a school applying integrated nursing curriculum. J Korean Acad Nurs Adm. (2008) 14:159–66.

[16] Facione NC Facione PA Sanchez CA. Critical thinking disposition as a measure of competent clinical judgment: the development of the California critical thinking disposition inventory. J Nurs Educ. (1994) 33:345–50. 10.3928/0148-4834-19941001-05 7799093

[17] Park BY Kim HG 2014 survey of citizens' perception of ODA. The Office for Government Policy Coordination Research Report. Gallup Korea (2014) Retrieved from http://www.odakorea.go.kr/mz.blltn.PolicySl.do?brd_seq=9&bltn_seq=177&print_no=45 (4 May 2019).

[18] World Research 2016 survey of citizens' perception of ODA. The Office for Government Policy Coordination Research Report. World Research (2016) Retrieved from http://www.prism.go.kr/homepage/theme/retrieveThemeDetail.do?leftMenuLevel=110&cond_brm_super_id=NB000120061201100059686&research_id=1092000-201700001 (4 May 2019).

[19] Bryar R Kendall S Mogotlane S Reforming primary health care: a nursing perspective. Contributing to health care reform, issues and challenges: ICN-International Council of Nurses (2011). p. 62

[20] Nelson BD Essential Clinical Global Health. ohn Wiley & Sons (2014).

[21] WHO (2010). Strategic directions for strengthening nursing and midwifery services 2011–2015 (No. WHO/HRH/HPN/10.1). Geneva World Health Organization Retrieved from http://whqlibdoc.who.int/hq/2010/WHO_HRH_HPN_10.1_eng.pdf. (accessed 4 May 2019)

[22] Maier CB Aiken LH. Expanding clinical roles for nurses to realign the global health workforce with population needs: a commentary. Isr J Health Policy Res. (2016) 5:21. 10.1186/s13584-016-0079-2 27280014 PMC4897947

[23] Nkowane AM Ferguson SL. The world health organization launches the 2016-2020 global strategic directions for strengthening nursing and midwifery. Nurs Econ. (2016) 34:206–7. Retrieved from https://search.proquest.com/docview/1812897547?accountid=11931 29975030

[24] Kim Y Han K Yoo HY. Enhancing undergraduate nursing students' global health competencies in South Korea. Public Health Nurs. (2017) 34:479–84. 10.1111/phn.12331 28488271

[25] Kang HR Hong Y Effects of international volunteering on global citizenship and multicultural acceptability among college students. J Civic Youth Stud. (2015) 6:1–39.

[26] Lee W (2012). Strategy for Globalization Capacity Building of Health Care Service Personnel. Retrieved from http://www.prism.go.kr/homepage/researchCommon/retrieveResearchDetailPopup.do?research_id=1351000-201200087 (accessed 4 February 2020)

[27] Kim YS Han M Convergence relationship between global citizenship, self leadership and global health competencies in nursing students. J Digit Convergence. (2018) 16:347–57.

[28] Osland JS Li M Petrone M Mendenhall ME Global leadership development in the university setting and future directions for advancing global leadership research. In: Advances in Global Leadership Vol. 11 Bingley: Emerald Publishing Limited (2019). p. 347–66.

[29] Huston C. Preparing nurse leaders for 2020. J Nurs Manag. (2008) 16:905–11. 10.1111/j.1365-2834.2008.00942.x 19094101

[30] Baik E Analysis of College Students' Awareness and Educational Needs on Global Leadership Competency by Global Experience Levels. Unbpublished doctoral dissertation, Hanyang University, Seoul (2015).

[31] Song YS A study on global leadership competencies of Korean enterprises. Korean J Hum Res Dev Q. (2011) 13:51–74. 10.18211/kjhrdq.2011.13.3.003

[32] Dawson M Gakumo CA Phillips J Wilson L. Process for mapping global health competencies in undergraduate and graduate nursing curricula. Nurse Educ. (2016) 41:37–40. 10.1097/NNE.0000000000000199 26164326

[33] Hur YJ A study on changes of university Leadership‘s curriculum for global leadership‘s key competencies. J Curric Stud. (2011) 29:235–64. 10.15708/kscs.29.4.201112.011

[34] Chun Y Kim K Hwang Y The effect short-term foreign volunteer activity of global leadership on university students. Asia-pac J Multimedia Serv Convergent Art Humanit Soc. (2017) 7:73–85. 10.14257/AJMAHS.2017.06.12

[35] Choi K Park Y The effects of team-based learning on problem solving ability, critical thinking disposition and self-directed learning in undergraduate nursing students. J East-West Nurs Res. (2014) 20:154–9. 10.14370/jewnr.2014.20.2.154

